# Effect of Dye Aggregation on the Sorption Behavior of Anionic Dyes onto Cationized Cellulose Fibers

**DOI:** 10.1021/acs.langmuir.5c02070

**Published:** 2025-07-08

**Authors:** Felix Netzer, Amalid Mahmud-Ali, Avinash P. Manian, Thomas Bechtold, Tung Pham

**Affiliations:** Research Institute of Textile Chemistry and Textile Physics, 27255 University of Innsbruck, Hoechsterstrasse 73, 6850 Dornbirn, Austria

## Abstract

The aim of this work was to investigate the sorption mechanism of anionic dyes (acid, reactive) at the liquid–solid interface between the dye solution and cationized cellulose. Viscose fibers were cationized with 3-chloro-2-hydroxypropyl-*N*,*N*,*N*-trimethylammonium chloride to a cationic group content of 25 to 113 mmol kg^–1^, and their sorption propensities were first examined in the absence of NaCl. With C.I. Acid Blue 25 and C.I. Reactive Red 120, the dye uptake followed a Langmuir isotherm at low dye concentrations, but at higher concentrations, an increase in dye uptake attributed to a nonionic sorption mechanism was observed. In the case of C.I. Reactive Blue 19, no nonionic sorption process occurred; rather, a decrease in dye uptake was observed. The latter is thought to occur due to the competitive sorption of anionic additives from the dye formulation on the cationic centers of the fiber. To gain a more comprehensive understanding of the processes involved, sorption equilibria were recorded in the presence of NaCl, together with assessments of the dye aggregation propensity in solution with ultraviolet–visible (UV/vis) spectrophotometry. A sorption model was developed using an adapted Langmuir–Sips isotherm that describes the sorption isotherm and accounts for dye aggregation in solution. Further, reactive dye fixation was investigated on a cationized cotton jersey to examine the transition from the sorption to the fixation stage in the dyeing process. The results of the work give insights into the adaptations required in dyeing recipes in the salt-free reactive dyeing of cellulosic materials.

## Graphical abstract

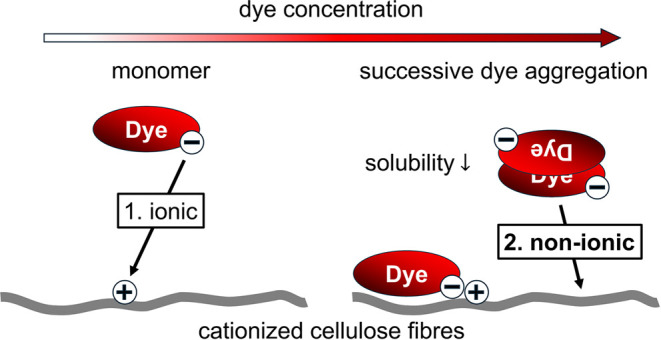

## Introduction

Cellulose fibers, both native and regenerated, account for more than one-third of the total textile fiber market and are the second most important fiber material after polyester fibers.^1^ Common to all cellulose textiles is that dyeing, especially reactive dyeing, is a very resource-intensive process, requiring large quantities of fresh water and additives in the form of salts, and producing saline and colored effluents.^2^ Alternative approaches, such as the cationization of cellulose are being investigated to reduce salt loads and dye content in effluents. These benefits are attributed to a change in the interactions (i.e., electrostatic attraction) between the anionic dye molecule and the grafted cationic group at the liquid–solid interface.

In a conventional dyeing process, electrostatic repulsion between the dye and fiber inhibits adsorption of dye molecules.^3^ The anionic charge of the fiber arises from ionization of hydroxy groups, which are an integral part of the cellulose structure and carboxyl groups, formed by oxidative processes during production of regenerated fibers^4^ or cotton pretreatments such as bleaching.^5^ Both groups are therefore inherent in a cellulosic textile fiber. Reactive dye molecules consist of a chromophore substituted by various groups, such as reactive anchor groups, which allow chemical fixation to the fiber, and anionic sulfonate groups for better solubilization.^6^ Therefore, electrostatic repulsive interactions may occur at the interface between the dye in solution and the fiber. Inorganic electrolytes, such as NaCl and Na_2_SO_4_, are added as charge screening agents to overcome the electrostatic repulsion and promote dye uptake. However, electrolyte amounts sufficient only for charge screening do not improve dye uptake, and so higher concentrations are required.^3,7^ This is attributed to the role of electrolytes in promoting self-association among dye molecules, which also increases dye uptake.

Dye molecules typically exhibit a long planar structure with extensive conjugated electron systems, which are prone to self-association of two or more molecules via π–π and van der Waals interactions.^8^ The tendency to self-associate depends, in addition to molecular structure, on the dye concentration, the temperature, the solvent, and the concentration of inorganic electrolytes or other additives.^9–11^ Inorganic electrolytes facilitate self-association due to charge screening effects between dye molecules resulting in the formation of dimers, trimers, and aggregates of higher order.^12^ In reactive dye solutions, the predominant observation is the formation of blue-shifted (hypochromic) H-dimers.^13^ In addition to this, H-aggregates and aggregates of higher order are also formed.^14^ It is important to understand that dye aggregation is highly relevant to reactive dyeing because the solubility of the dye molecule is lower in its aggregated form and therefore the tendency to adsorb onto the fiber is increased.^15^ The exact mechanism by which sorption occurs, i.e., if individual molecules from aggregates or the whole aggregate adsorbs to the fiber and disaggregates is not fully understood.^7^

Mechanistically, the sorption phenomena occurring at the dye solution–fiber interface are different for cationized and noncationized fibers,^16^ as shown schematically in Figure 1. In cationized fibers, sorption is governed by an ionic interaction at the dye solution–fiber interface (A), and the dye molecules are expected to adsorb onto or in close proximity to the cationic group.^17,18^ Nonionic interactions, such as H-bonds or van der Waals interactions, are secondary and contribute to the total binding strength of the molecule to the fiber. This sorption process is typically described by the Langmuir model,^19–21^ which assumes that all available sorption sites have the same energy, the adsorption is limited by the number of sorption sites, and there are no interactions between sorbates.^22^

**Figure 1:**
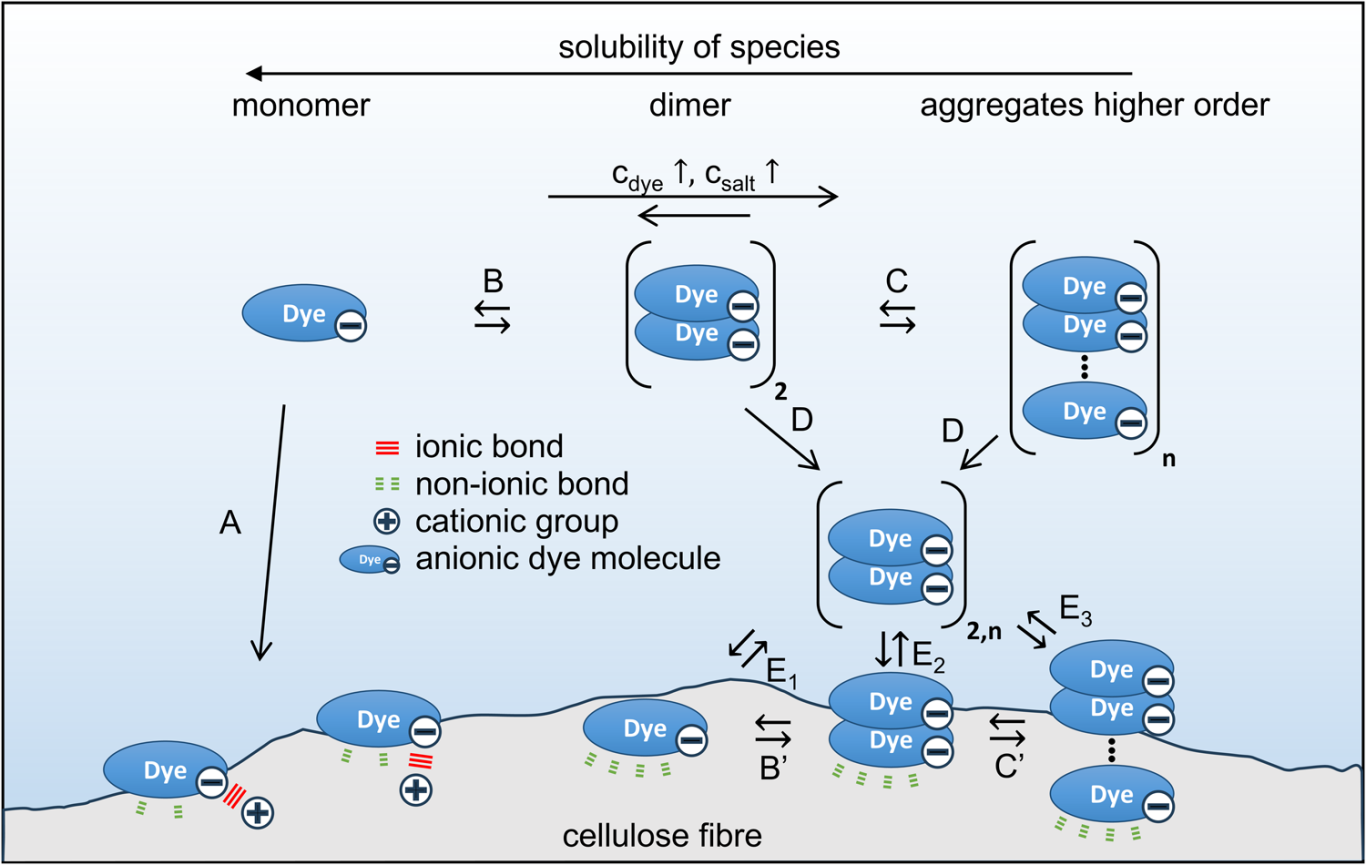
Schematic representation of the sorption phenomena at the dye solution–fiber interface for cationized and noncationized cellulose fibers. For cationized fibers, the main mechanism is the adsorption of a monomer onto a cationic group due to ionic interactions (A). For untreated fibers, the addition of salt and higher dye concentration results in the formation of dimers (B) and higher-order aggregates (C). These species have lower solubility (D) and adsorb onto the fiber as individual molecules (E_1_), dimers (E_2_), or aggregates (E_3_), due to nonionic interactions. The adsorbed molecules may aggregate or disaggregate (B′ and C′) on the fiber.

In noncationized cellulose, dye sorption is promoted by the presence of inorganic electrolytes inducing self-association of dye molecules to aggregates (B and C) with lower solubility compared to the monomer (D). Sorption occurs due to nonionic interaction between the dye and fiber, whereby the dye molecules may adsorb as a monomer (E_1_), dimer (E_2_), or higher-order aggregate (E_3_) and may aggregate or disaggregate (B′ and C′) on the fiber.^7,23,24^ This process is described by the Freundlich model, where there is no restriction in adsorption to specific sites, and an infinite number of heterogeneous sorption sites with different sorption energies are available.^25^ It should be noted that the present discussion is limited only to the first stage of reactive dyeing (sorption stage), prior to the addition of alkali to induce covalent fixation.

In recent literature concerning cationization and salt-free reactive dyeing, a significant amount of research has been conducted on the enhancement of the process.^20,26,27^ For instance, the impact of the cationization level^28,29^ or cationization agent^19,30–32^ on the outcome of reactive dyeing was investigated. Another issue that must be addressed is the controllability of the dyeing process, as some dyes are prone to uneven dyeing.^33,34^ It has been reported in other studies that this process is feasible in improving the ecological profile of reactive dyeing. This has been demonstrated in pilot-scale experiments.^35–37^ A considerably smaller number focused on the interaction between anionic dye molecules and cationized cellulose fibers.^16,38^ In previous work,^39^ the potential of using low-cationization levels for reactive dyeing was investigated with the goal of reducing the chemical input required during cationization, thereby improving cost-effectiveness for industrial scale. Although this earlier study examined the same dyeing process, it was conducted using model systems with highly diluted dye solutions, which differ significantly from the higher dye concentrations typically used in industrial applications.

Since dye aggregation is a function of dye concentration and plays a fundamental role in reactive dyeing, the present study examines the impact of dye aggregation on dye sorption on cationized cellulose fibers.

## Experimental Section

### Materials and Chemicals

Loose viscose fibers of type Danufil F (dTex of 1.7; staple length of 40 mm), which exhibited an inherent carboxyl group content of 68 mmol kg^–1^ as measured previously, were kindly donated by Kelheim Fibers GmbH (Germany).^40^ Fibers were used in the investigation on sorption mechanism as it allowed for optimum accessibility to reagents during cationization and dye sorption. Mercerized and ready-to-dye cotton jerseys (areal weight of 130 g m^–2^) were kindly provided by a local textile processing company and used for dye fixation investigations as it allowed for mimicking an industrial operation and also for assessment of dyeing levelness (i.e., uniformity of color appearance across a surface). CHPTAC (3-chloro-2-hydroxypropyl-*N*,*N*,*N*-trimethylammonium chloride) was used as a cationization agent and purchased as 60 wt % solution from Sigma-Aldrich. The dyes studied, C.I. Acid Blue 25 (AB25; purity = 45 wt %; MW = 416.4 g mol^–1^), C.I. Reactive Blue 19 (RB19; purity = 42 wt %; MW = 626.5 g mol^–1^), and C.I. Reactive Red 120 (RR120; purity = 55 wt %; MW = 1470.0 g mol^–1^), were purchased from Sigma-Aldrich. The dye structures are listed in Figure 2. Collasol SD, a nonionic wetting agent used in the cationization of the jersey was from CHT Switzerland AG. Microbiological grade agar was used in sample preparation for nitrogen analysis and HPLC grade l-aspartic acid (*N* content = 10.52 wt %) was used in calibration of the device. All other chemicals, including sodium hydroxide, sodium carbonate, sodium acetate, acetic acid, hydrochloric acid, sodium chloride, and dimethylformamide (DMF), were at minimum, of reagent grade. Unless otherwise stated, deionized H_2_O with a conductivity of less than 0.2 μS cm^–1^ was used in all experiments, and all chemicals were used without further purification.

**Figure 2:**
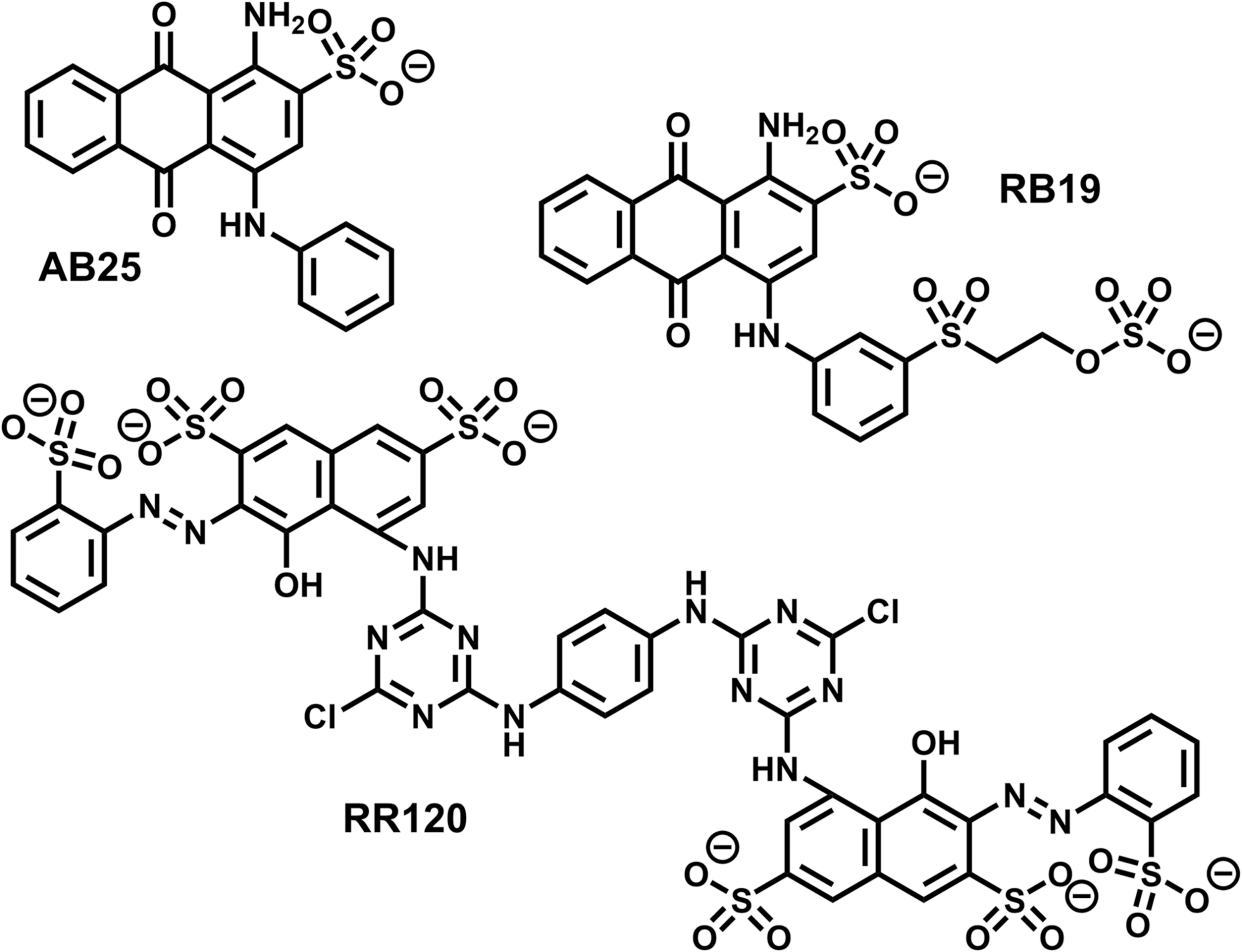
Molecular structure of the investigated dyes.

### Demineralization and Cationization of Viscose Fibers

These treatments of loose viscose fibers followed a previously reported routine^41^ of two successive treatments, demineralization and cationization, with a drying step in between. Demineralization was carried out to remove all spin finishes and minerals from the fiber, by immersing in an aqueous HCl solution (1 wt %, 1 g/35 mL, 40 °C) for 1 h, followed by two rinses with deionized water to remove excess HCl and neutralization with an aqueous sodium acetate solution (1 g L^–1^, 1 g/35 mL, 40 °C) for 30 min. After three rinses with deionized H_2_O, the fibers were air-dried and stored for further use. Cationization was carried out with CHPTAC and sodium hydroxide at a molar ratio of 1:1.8. The fibers were first placed in a CHPTAC solution (1 g/15 mL, 20–22 °C) and agitated on a roller shaker for 30 min to achieve uniform wetting of the fiber. Aqueous NaOH was added to give a final liquor ratio of 1 g/20 mL, followed by further 30 min of agitation on the roller shaker. The reaction mixture was then placed in a shaking water bath and heated from room temperature to 70 °C within 30 min. This temperature was maintained for 60 min before the fibers were placed in a sieve, rinsed with deionized water, and immersed in acetic acid solution (0.5 wt %, 70 °C) for 30 min. In addition, the fibers were washed twice more with acetic acid solution and three times with deionized water before being dried in air. The compositions of the cationization solutions are given in Table 1.

**Table 1:** Composition of the Cationization Solution, the Cationic Group Content (*N* ^+^), the Cationic-to-Carboxyl Group Ratio, and the Reaction Yield of the Cationized Samples Investigated in This Work

		CHPTAC		NaOH		N^+^ (mmol kg^–1^)	N^+^:COO^–^ ^a^	yield (%)
sample		g L^–1^	mmol L^–1^	g L^–1^	mmol L^–1^			
fiber	A	5.8	31	2.2	55	48 ± 5	0.7	7.8 ± 0.8
fiber	B	7.7	41	3.0	75	68 ± 7	1.0	8.7 ± 0.5
fiber	C	9.6	51	3.7	93	93 ± 3	1.4	9.1 ± 0.3
fiber	D	11.6	62	4.4	110	113 ± 5	1.7	9.2 ± 0.4
fiber	E	4.4	23	1.7	43	31 ± 4	0.3	6.6 ± 0.9
fiber	F	7.0	37	2.7	68	57 ± 4	0.8	7.7 ± 0.5
fiber	G	9.6	51	3.7	93	93 ± 12	1.4	9.1 ± 1.2
jersey	H	34.0	181	12.9	323	46 ± 10	n/a	32.0 ± 0.3

a The carboxyl-to-cationic group ratio is dimensionless.

### Cationization of Cotton Jersey

The mercerized and ready-to-dye cotton jersey was cationized in a cold pad batch process. Soft water instead of deionized water was used for cationization and washing. The fabric was immersed in a solution containing CHPTAC and NaOH in a molar ratio of 1:1.8 and 1 g L^–1^ wetting agent Collasol SD. By passing the jersey through a padding mangle, a wet pick-up of 80% was achieved. The impregnated jersey was wound onto a roll, wrapped in a plastic film, and left to rest for 20 h under continuous rotation. The cationized jersey was then rinsed once with H_2_O, washed with 0.01 wt % acetic acid at room temperature, followed by a hot wash with 0.02 wt % acetic acid at 80 °C, and a final wash with water at room temperature.

### Nitrogen Analysis

The degree of cationization was determined by nitrogen analysis using a rapid N III nitrogen analyzer (Elementar Analysensysteme GmbH, Germany). Carefully weighed samples of approximately 350 mg were wrapped in tin foil, placed in a 3D-printed punch cell, pressed into tablet form, covered with hot agar gel (2.5 wt %), and centrifuged at 4000 G to remove air trapped in the sample tablets. After solidification of the agar gel, the tablets were placed in the testing apparatus to determine the nitrogen content (details in the Supporting Information). The percentual nitrogen content was determined from a linear calibration curve obtained with l-aspartic acid as a standard (Figure S2).

The molar nitrogen content was calculated according to eq 1, where *N* ^+^ is the molar nitrogen content (mmol kg^–1^), *N*% is the percentual nitrogen content, and *A*_r_(*N*) is the atomic weight of nitrogen (14.0067 g mol^–1^).

1 $$N^{+}=\frac{N\%}{A_{r}\left(N\right)}\cdot 10^{4}$$

### Sorption Experiments

The sorption behavior of anionic dye molecules on cationized cellulose fibers was investigated by a series of sorption experiments at neutral pH 5–7 using aqueous solutions of AB25, RB19, and RR120 in capped glass bottles to prevent evaporation of the solution.

*Sorption isotherms* were obtained by immersing 0.5 g of the cationized and reference fibers in 200 mL of dye solution with concentrations ranging from 0.025 to 1.2 g L^–1^. For the cationized samples, no NaCl was added, and for the untreated reference fibers 50 g L^–1^ NaCl was added. A shaking water bath at 30 °C was used to ensure constant temperature and adequate agitation of the liquid and fibers. After 24 h, the residual dye bath was analyzed by ultraviolet–visible (UV–vis) spectrophotometry in the range 350–700 nm (model: MCS 601, Carl Zeiss Spectroscopy GmbH, Germany), allowing the concentration in the dye bath to be calculated from linear calibration curves of the individual dyes. Finally, the dyed fibers were washed and rinsed with water and allowed to air-dry. The experiments were conducted in triplicate for all dye concentrations and cationization levels. The experimental data of the isotherm were fitted to the Langmuir and Freundlich models. These models are described by eqs 2 and 3, where *q*_e_ is the equilibrium sorption (mol kg^–1^), *c*_e_ is the equilibrium concentration (mol L^–1^), *S*_L_ is the monolayer sorption (mol kg^–1^), *K*_L_ is the Langmuir constant (L mol^–1^), *K*_F_ is the Freundlich constant (mol kg^–1^) and *n* is the Freundlich exponent.

2 $$q_{e}=\frac{S_{L}\cdot K_{L}\cdot c_{e}}{1+K_{L}\cdot c_{e}}$$

3 $$q_{e}=K_{F}\cdot c_{e}^{n}$$

*Sorption equilibria in the presence of NaCl* were obtained by immersing 0.5 g of fibers, both cationized and untreated, in 50 mL of a 0.4 g L^–1^ dye solution of RB19 and RR120. AB25 was excluded from these experiments, as it readily flocculated when NaCl was added. The dye solution was without NaCl or with 5, 25, and 50 g L^–1^ NaCl. After a sorption phase of 24 h with agitation in a tempered water bath at 30 °C, the residual dye solution was measured by UV/vis spectrophotometry, and the concentration was determined from linear calibration graphs for each dye and salt levels. The experiments were performed in triplicate.

In preliminary tests, it was observed that a total amount of 20 mg of dye was sufficient to approach a sorption plateau on the cationized samples. To minimize the effects of dye aggregation and to investigate the ionic sorption mechanism, the liquor ratio was set at 1:400, which allowed the plateau to be observed in a 0.1 g L^–1^ (20 mg in 200 mL) dye solution for sorption isotherm experiments. Conversely, in salt-assisted sorption equilibria, the liquor ratio was reduced to 1:100 to increase the chances of dye self-association (0.4 g L^–1^, 20 mg in 50 mL).

### Dye Fixation Experiments

Reactive dyeing experiments were conducted to investigate the transition from the sorption phase to the fixation phase. The addition of alkali disrupts the sorption equilibria by ionizing cellulose hydroxy groups and fixing reactive dyes. Cationized and untreated cotton jerseys were subjected to salt-free and conventional dyeing experiments with 50 g L^–1^ NaCl. About 4 g of jersey was immersed in 35.4 (or 33.8) mL of water and allowed to equilibrate at 40 °C for 10 min before 1.6 mL of 25 g L^–1^ (or 3.2 mL of 12.5 g L^–1^) dye solution was added. In the case of the conventional dyeing process, the dye bath contained 50 g L^–1^ NaCl. After 5 min, the temperature was increased to 60 °C within 20 min and held for a further 10 min before a total of 3 mL of 66.7 g L^–1^ Na_2_CO_3_ solution was added in five doses over a period of 20 min. The volume of the first two doses was 0.375 mL each, and the volume of the other three doses was 0.75 mL each. The reaction was continued for 55 min, giving a total dyeing time of 2 h. The dyed jerseys were rinsed with deionized water and washed five times for 10 min, each with a volume of 40 mL, in water, 0.5 wt % acetic acid, both at room temperature, followed by a hot wash at 95 °C and two cold washing steps at room temperature, all three with water. The washed jerseys were allowed to air-dry.

### Analysis of Dyed Samples

*Dye extraction* experiments were performed to test for dye fixation. The dyed samples were immersed in a 50 vol % DMF solution containing 50 g L^–1^ NaCl at 100 °C for 30 min.

Investigation of the color appearance of the washed samples from the isotherm and fixation experiments were carried out by *reflectance spectrophotometry* using a d/8 spectrophotometer equipped with a pulsed xenon lamp as a light source (model: CM 3610d, Konica Minolta, Japan). Three (fibers) or six (jersey) measurements were taken per sample to record the specular component excluded reflectance curve. Color strength was calculated using the Kubelka–Munk equation at a single wavelength (640 nm for AB25, 600 nm for RB19, and 550 nm for RR120). In the equation, *K* and *S* are the absorption and scattering coefficients, respectively, and *R*_∞_ is the fractional reflectance (eq 4).

4 $$K/S=\frac{1-R_{\infty }}{2R_{\infty }}$$

### Absorbance Spectroscopy

The dye aggregation propensities were determined from absorbance spectra of AB25, RB19, and RR120 recorded in 70 vol % ethanol, deionized H_2_O, and 5, 25, and 50 g L^–1^ NaCl at concentrations of 0.05, 0.1, 0.2, 0.5, and 1 mmol L^–1^ dye. Measurements were taken using a 0.1 mm quartz glass flow cell. The monomer/dimer ratio was determined for each absorbance spectra and normalized by the ratio obtained in EtOH/H_2_O. This was done according to eq 5, where *r* is the ratio of monomer to dimer and *A*_mono_(*c*_i_) and *A*_dimer_(*c_i_*) are the absorbance maxima for the monomer and dimer peaks at concentration *c_i_*, respectively.

5 $$r_{i}^{normalized}=\frac{r_{i}}{r_{i}^{EtOH:H_{2}O}}=\frac{A_{mono}\left(c_{i}\right):A_{\dim er}\left(c_{i}\right)}{A_{mono}^{EtOH:H_{2}O}:A_{\dim er}^{EtOH:H_{2}O}}$$

## Results and Discussion

### Cationization Reaction

CHPTAC was chosen as the cationization agent because of its ease of application and small size, which makes it an ideal model substance to study ionic charges on the fiber without significant contribution from the molecular structure of the agent. The mechanism of reaction between cellulose, CHPTAC, and NaOH is well documented in the literature and is shown in the Supporting Information (Figure S3). In Table 1, information on cationization and the investigated samples is found. A cationic group content of 25 to 113 mmol kg^–1^ corresponds to a relatively low level of cationization compared to much higher levels reported in the literature.^21,42^ Reaction yields of up to 9% for viscose fibers (exhaust) and 32% for cotton jersey (cold pad batch) are in line with expected reaction yields for cationization with CHPTAC in these processes.^43^ It should be noted that fibers C and G have a similar cationic group content; however, they are treated as two separate samples, as they were obtained from different batches.

### Sorption Isotherms

Isotherms were studied on four samples (fibers A and D) where the cationic group content was lower, similar, or higher than the inherent carboxyl group content. Figure 3 shows sorption equilibrium data in the form of salt-free sorption isotherms of AB25 (a), RB19 (b), and RR120 (c) and sorption isotherms on noncationized cellulose with the addition of 50 g L^–1^ NaCl (d). From the sorption isotherms on cationized samples, it can be seen that the equilibrium dye uptake increased both with the degree of fiber cationization and dye concentration in the bath, but differences were observed between the three dyes. At low initial dye concentrations in bath (i.e., <0.0625 g L^–1^ or 0.038 mmol L^–1^), complete exhaustion of AB25 was observed (Figure 3a), resulting in isotherms showing an initial near-vertical increase in dye uptake followed by a sharp transition to a well-defined plateau. This segment correlates very well with the Langmuir model (*R* ^2^ ≈ 0.95–0.98) and less so with the Freundlich model (*R* ^2^ ≈ 0.89–0.96). For RR120 (Figure 3c), similar sorption curves are obtained as for AB25, with the only notable difference being a more gradual transition to a plateau. Again, the Langmuir and Freundlich models show very good (*R* ≈ 0.96–0.99) and not so good (*R* ^2^ ≈ 0.79–0.97) correlations with the experimental data. However, for both dyes, as the dye concentration in the bath increased further, an increase of dye uptake is observed, which is not consistent with either Langmuir or Freundlich model. The increase may be a result of nonionic interactions between dye molecules and the fiber, similar to a conventional sorption process on noncationized cellulose. For RB19 (Figure 3b), dye uptake initially increases almost vertically with dye concentration up to a maximum, but beyond that a reduction of dye uptake is observed rather than a plateau. Such observation has been reported previously by other authors^44^ and could be attributed either to dye desorption from fibers or some aspect that interferes with proper measurement of changes in dye concentration in liquors such as dye precipitation or loss of volume due to evaporation. The stability of dye in liquor was assessed by measuring the absorbance of a dye solution maintained at 30 °C over time (7 days) under similar experimental conditions, and no significant changes were observed; and as the dyeing vessels were capped bottles, significant changes to dye volumes through evaporation are not expected. Therefore, it may be accepted that there was desorption of dye from fibers. Desorption could occur if the conditions of the system, such as the temperature and pH, changed. Temperature variations were prevented by using a tempered water bath, and no pH changes were observed before and after the experiment. Another aspect to consider is that the exact composition of the dye formulation is largely unknown. It is common practice to incorporate ionic additives^45^ which, in the case of an ion-exchange process, could result in a competitive sorption behavior between the fast-adsorbing dye and a slower but more strongly binding anionic additive. Thus, dye uptake is lowered with increasing dye concentration as the ionic component increased concomitantly. This trend is maintained at higher dye concentrations, except at the lowest level of cationization. Here, a slight increase in dye uptake is observed, which is attributed to the low number of cationic sites for an exchange mechanism and nonionic binding of dye molecules at higher concentrations. As shown in Figure 3b, the data for RB19 cannot be satisfactorily fitted to the Langmuir model.

**Figure 3:**
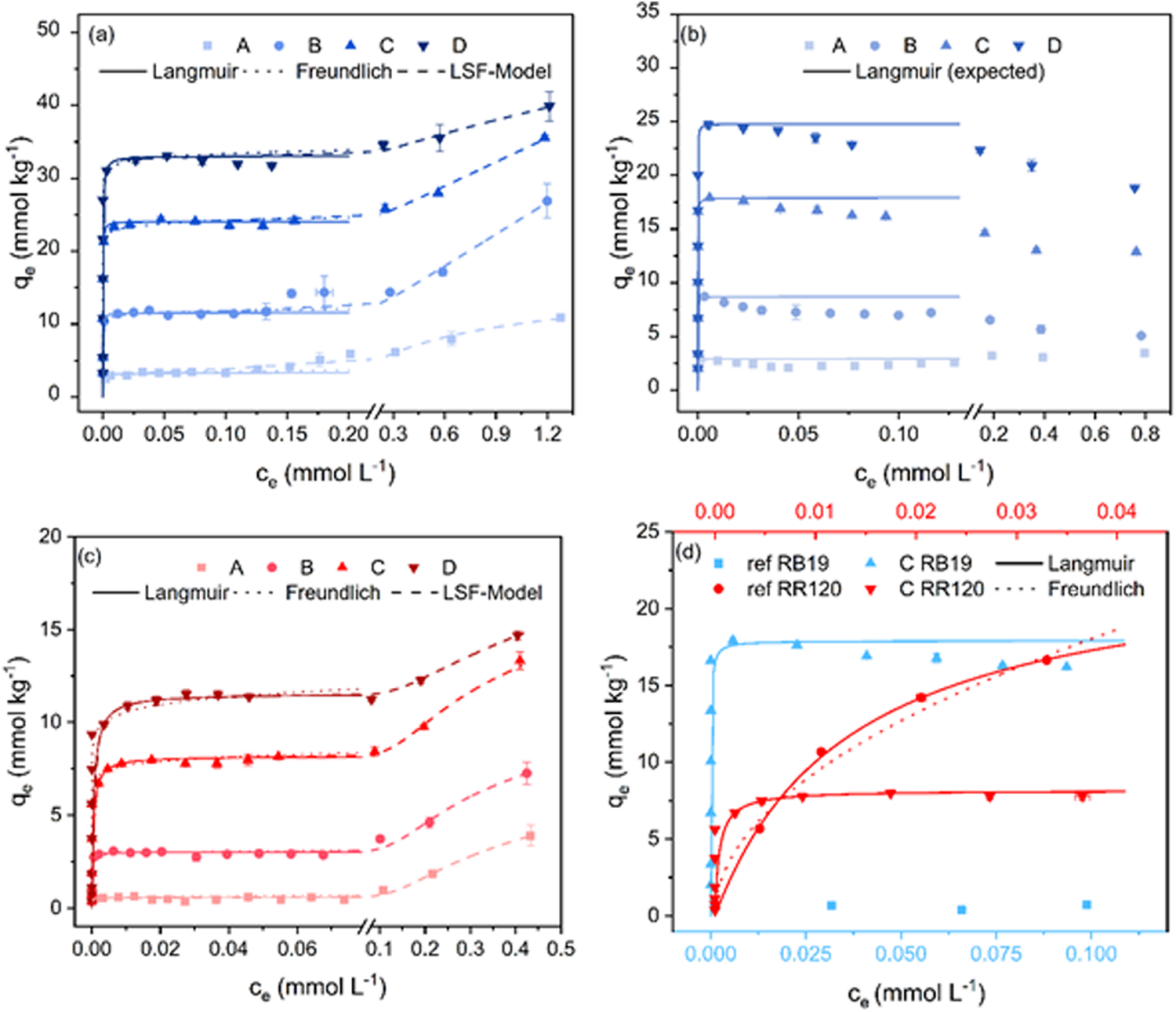
Sorption isotherms for AB25 (a), RB19 (b), and RR120 (c) on cationized fibers. In (d), the sorption isotherms of fiber C obtained from the salt-free dyeing experiment with RB19 and RR120 are compared with the sorption data of an untreated reference fiber with 50 g L^–1^ NaCl added to the dye bath. The upper *x*-axis represents the equilibrium concentration of RR120 and the lower *x*-axis that of RB19. It should be noted that the *y* and *x* axes are scaled differently for each graph.

In Figure 3d, the salt-free sorption curves (exemplarily shown for fiber C) of RB19 and RR120 are compared with the sorption curves of an untreated reference fiber immersed in dye solution also containing 50 g L^–1^ NaCl. It can be observed that the uptake of RR120 is higher with salt on the reference fiber as compared to without salt on the cationized fiber, whereas the reverse is true for RB19. This suggests that RB19 sorption is more sensitive to the presence of cationic centers on fibers and less to the presence of salt, and that the investigated level of cationization has a very limited impact on RR120 uptake and the presence of salt exerts a far greater impact. The very low uptake of RB19 on reference fibers in the presence of salt is unexpected, and perhaps greater salt levels would be required to induce dye sorption. For AB25, it is not possible to record reference sorption curves due to the fact that AB25 starts to flocculate with minor additions of NaCl (<5 g L^–1^).

Dye aggregation is promoted by the addition of inorganic electrolytes and by increasing the dye concentration in solution.^24,46^ This consideration is used to explain the salt-free sorption isotherms in Figure 3. Once the Langmuir plateau is approached, no further dye uptake occurs until the dye concentration is high enough to initiate sufficient self-association. As a result of the lower solubility of aggregates, an increase in dye uptake is observed for AB25 and RR120. The first sorption process is governed by an ion-exchange mechanism resulting in a Langmuir-type isotherm, whereas the second sorption process is characterized by nonionic and noncovalent interactions. Therefore, the second rise in dye uptake represents the mechanism observed in conventional reactive dyeing, and the sorption profiles are typically characterized by Freundlich isotherms. The occurrence of both sorption mechanisms on cationized cellulose is attributed to the rather low degree of cationization, where every 50th to 100th anhydrous glucose unit is cationized and there is a high residual dye concentration, which is necessary for dye aggregation. The lack of a second rise in RB19 uptake suggests a lower tendency to self-associate, which is discussed in greater detail in later sections.

Langmuir isotherms with a pronounced plateau at low concentrations and an increase in sorption levels at higher concentrations were reported, i.e., by Gago et al.,^47^ who observed that methylene blue sorption on dicarboxymethylated cellulose follow a Sips isotherm. The Sips isotherm, also known as the Langmuir–Freundlich isotherm (eq 6), is a combination of the Langmuir and Freundlich models, with the Freundlich model describing the first part of the curve and the Langmuir model describing the appearance of the plateau at a higher concentration. Lee et al.^48^ studied the adsorption of gas molecules on a porous surface, which exhibited a Langmuir plateau at low pressure and an increase in gas adsorption at higher pressure due to capillary condensation. A hybrid Langmuir–Sips (LS) isotherm (eq 7) describing a Langmuir plateau at a low concentration and an S-shaped increase in sorption with a second plateau at a higher concentration was used to fit the data. Fitting the LS equation to the sorption data of AB25 and RR120 is possible and shows good correlation (*R* ^2^ > 0.96) for some isotherms but results in a second sorption plateau, which is not the expected outcome for this sorption process. With a small adaptation of the LS model and adding the Freundlich expression to the second part of the equation, it is possible to obtain a mathematical model that describes a Langmuir sorption plateau at low concentrations, a Sips-related expression to account for successive dye aggregation, and a Freundlich term to describe dye uptake once aggregation has occurred (eq 8). This isotherm, here called LSF (Langmuir–Sips–Freundlich), describes the sorption data over the full range with good correlation for all isotherms (*R* ^2^ >0.97).

6 $$q_{e}=\frac{S_{L}\cdot K_{S}\cdot c_{e}^{n}}{1+K_{S}\cdot c_{e}^{n}}$$

7 $$q_{e}=S_{L}\cdot \left(\frac{K_{L}\cdot c_{e}}{1+K_{L}\cdot c_{e}}+\frac{K_{S}\cdot c_{e}^{n}}{1+K_{S}\cdot c_{e}^{n}}\right)$$

8 $$q_{e}=\frac{S_{L}\cdot K_{L}\cdot c_{e}}{1+K_{L}\cdot c_{e}}+\frac{K_{A}\cdot c_{e}^{n_{A}}}{1+{K_{A}\cdot c}_{e}^{n_{A}}}\cdot \left(K_{F}\cdot c_{e}^{n}\right)$$

Here, *q*_e_ is the dye uptake (mmol kg^–1^); *c*_e_ is the residual concentration of the dye bath (mmol L^–1^); *S*_L_ is the monolayer absorbance (mmol kg^–1^); *K*_S_, *K*_L_, and *K*_F_ are the constants (L mmol^–1^); *n* is the dimensionless Freundlich exponent. The parameters *K*_A_ (L mmol^–1^) and *n*_A_ (dimensionless) account for dye aggregation, where *K*_A_ is related to the dye concentration at which aggregation begins to promote dye uptake and *n*_A_ is related to the intensity with which aggregation affects dye uptake. The best fits were obtained by using the *K*_L_ and *S*_L_ parameters obtained from the Langmuir fitting of the plateau region and by fitting *K*_A_ and *n*_A_ as global parameters to the four isotherms at different cationization levels for each dye and *K*_F_ and *n* as individual parameters to the whole data range of each isotherm. The differences in fitting of the experimental data between the Langmuir, Freundlich, LS, and LSF models are shown in Figure 4. It should be noted that the dye sorption at the two highest dye concentrations in solution were obtained from experiments with a material/liquor ratio of 1 g:100 mL. This was because with high dye concentrations at the original ratio of 1 g:400 mL, it is difficult to detect small differences in dye sorption values.

**Figure 4:**
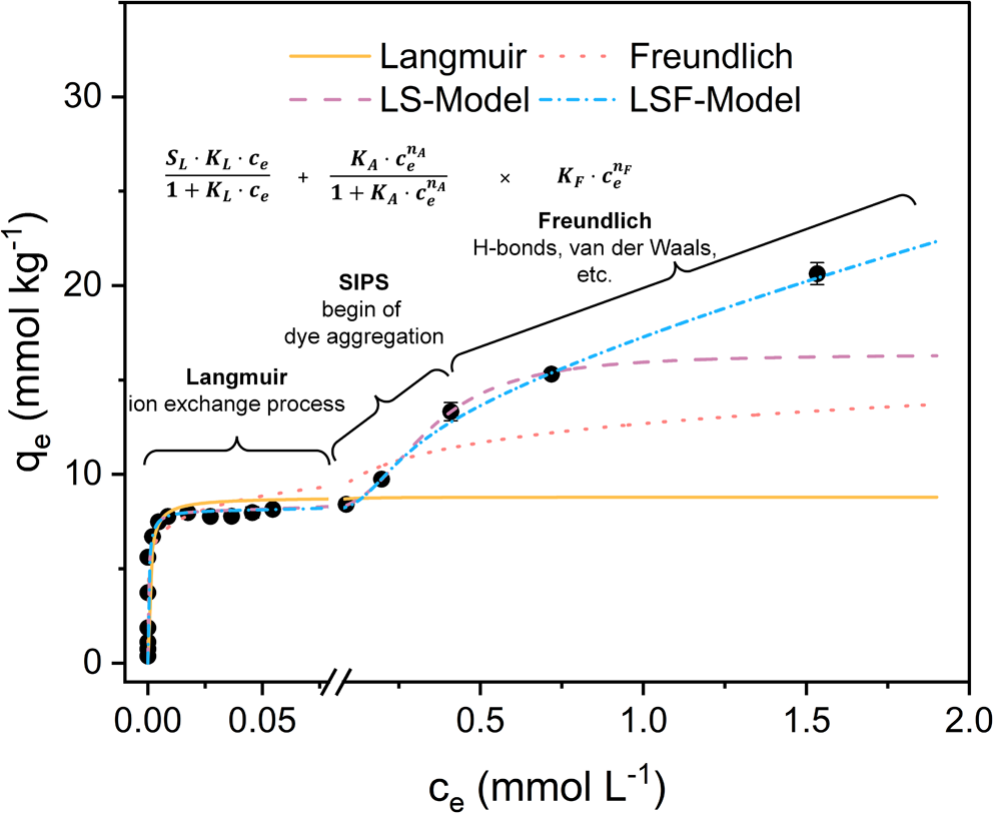
Comparison of the simple Langmuir and Freundlich equations with the hybrid LS and LSF models to describe the sorption data of RR120 on fiber C. Only the LSF model is able to describe the experimental data with excellent correlation (*R* ^2^ > 0.99).

### Reflectance Measurements

As a complementary method to substantiate the result from the isotherms, reflectance measurements were carried out on the thoroughly washed and dried fibers to determine the color strength. There is typically a linear correlation between the dye uptake and the *K*/*S* values at the wavelength of maximum intensity.^39^ Figure 5 plots the *K*/*S* values of the dyed fiber against the final dye concentration, both obtained from the sorption equilibrium experiment.

**Figure 5:**
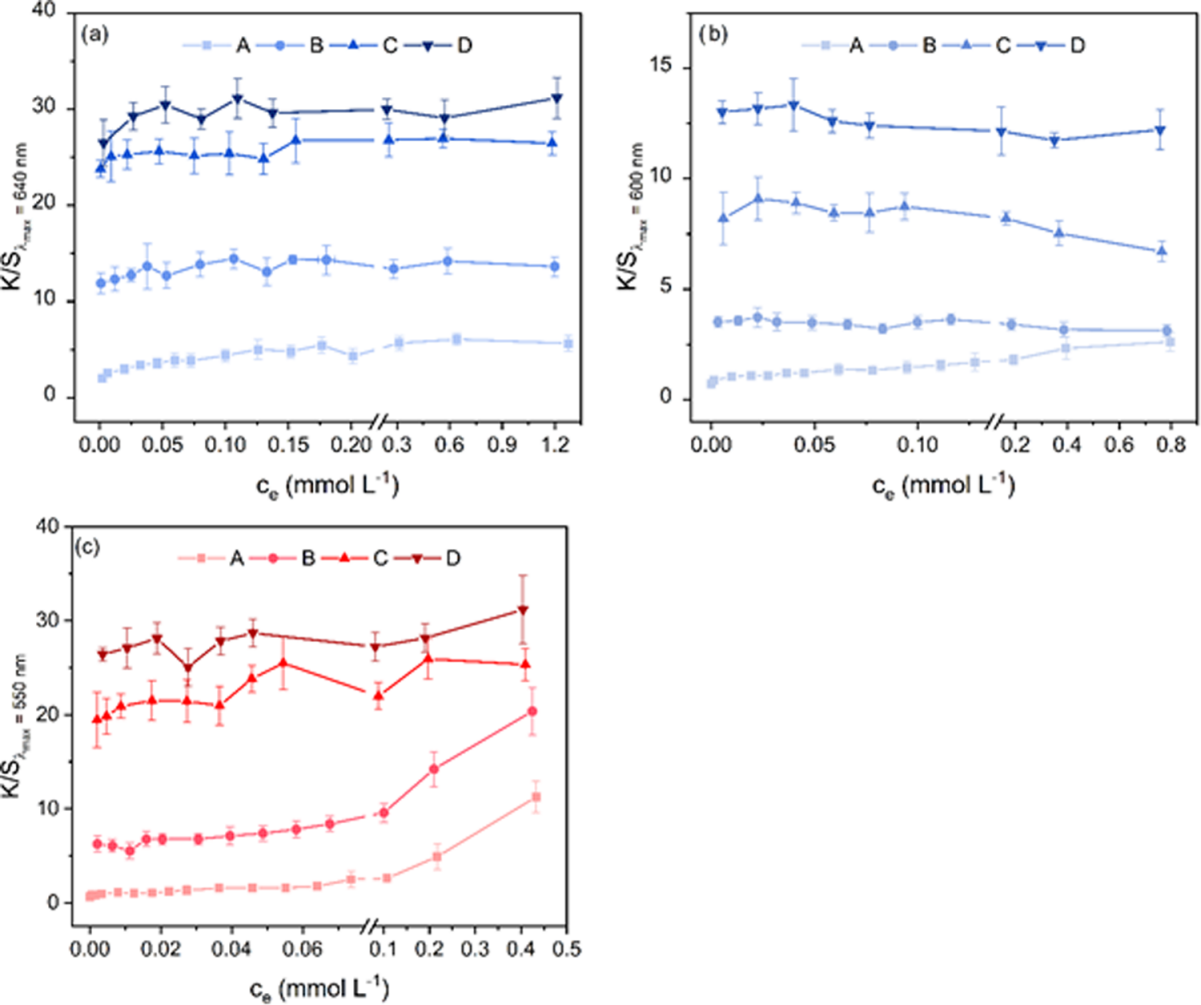
Color strength of AB25 (a), RB19 (b), and RR120 (c) dyed fibers as a function of the equilibrium concentration. Both the fiber and residual dye concentration were obtained from the isothermal sorption experiments.

For RR120 (Figure 5c), it can be seen that the trend of the curve reflects the trend of the sorption isotherm as a plateau and a second rise in dye uptake are well indicated. This is more evident for the lower cationization levels compared to the higher cationization levels, as the validity of the linear correlation decreases at *K*/*S* values of 20–25, since the intensity of the reflectance falls below 2% lowering the sensitivity to changes. In the case of RB19 (Figure 5b), the observed desorption of dye molecules is corroborated by a decrease in the *K*/*S* values. For the lowest cationization level, the divergent trend of the isotherm plot is confirmed by an increase in the *K*/*S* values. In contrast, the *K*/*S* values of AB25 (Figure 5a) remain at the plateau level and do not show an increase even though increased dye uptake was observed in the sorption experiments. This may be because of that the dye washed off when fibers were rinsed after sorption experiments, attributed to a weaker nonionic dye–fiber interaction for AB25 as compared to RR120.

### Sorption Equilibria in the Presence of NaCl

To further understand the impact of self-association of dye molecules on dye sorption, the sorption equilibria on cationized cellulose fibers in the presence of varying amounts of NaCl were examined. Viscose fibers with a cationic group content significantly lower (E), moderately lower (F), or higher (G) than the carboxyl group content and a noncationized reference were used in this experiment and subjected to 24 h sorption tests in dye solutions of 0.4 g L^–1^ without or with 5, 25, or 50 g L^–1^ NaCl (Figure 6). Fibers E, F, and G were prepared for this experiment to ascertain how very low-, moderate-, and high-cationization levels, relative to the carboxyl group content, perform when salt is added.

**Figure 6:**
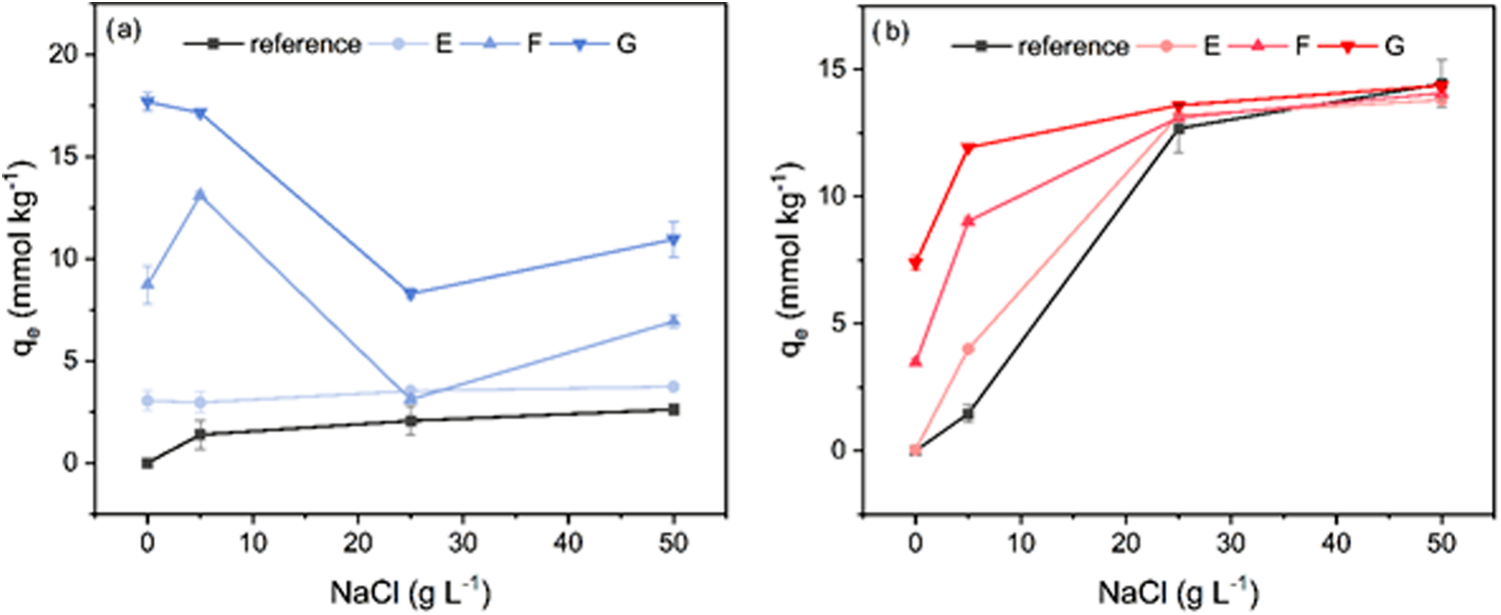
Sorption levels of RB19 (a) and RR120 (b) at different cationization levels as a function of the NaCl concentration in the dye bath.

For RB19 (Figure 6a), the dye uptake increased significantly with an increase in degree of cationization, but increasing the salt concentration in the dye bath had either no impact or a deleterious effect on dye sorption levels. A positive influence of cationization was observed on RR120 sorption levels (Figure 6b), but the addition of salt exerted greater influence on dye sorption. At salt contents in a bath of 25 g L^–1^ and higher, all fibers exhibited very similar dye sorption levels regardless of the cationization level.

To understand sorption equilibria in salt-assisted dyeing, it is important to note that the role of NaCl in the sorption process of anionic dyes on cationic fibers is a combination of charge screening, competitive ion exchange (IEX), and promotion of dye aggregation, occurring to different extents. Charge screening of anionic charges from the dye and ionic charges (carboxyl + cationic groups) from the fiber occurs at rather low NaCl levels.^7^ A competitive ion-exchange process starts at low NaCl concentrations but becomes more pronounced at higher concentrations. Significant dye aggregation starts at medium to high NaCl concentrations (20–50 g L^–1^) but always depends on the structure of the dye and its tendency to self-associate. These considerations can be observed in the differing sorption behaviors of RB19 and RR120 on cationized cellulose upon addition of NaCl. For RB19, the sorption behavior can be explained by the contribution of all three mechanisms. At low levels of cationization (fiber E), the low number of cationic sites limits the competitive IEX process to a minimum; thus, an increase in dye uptake is attributed to charge screening and dye aggregation at higher NaCl contents. For fiber F, the increase in dye uptake at 5 g L^–1^ NaCl is attributed to charge screening, the lower dye uptake at 25 g L^–1^ to competitive ion-exchange processes, and the increase at 50 g L^–1^ to dye aggregation. Similar observations were made for the adsorption of Cibacron Red HF on cationized cotton in the presence of Na_2_SO_4_.^49^ For fiber G, the high level of cationization makes the fiber more susceptible to competitive ion exchange even at low NaCl concentrations. In the case of RR120, the higher affinity and the high tendency to dye aggregation result in an increase in dye uptake at all cationization levels as the NaCl content is increased.

### Absorbance Spectra of Dyes

In UV/vis spectrophotometry, changes in the shape of the absorbance curve, such as peak position, peak height, or peak broadening, are indicative of dye aggregation. Figure 7 shows the normalized absorbance curves of AB25 (Figure 7 a), RB19 (Figure 7b), and RR120 (Figure 7c). In EtOH/H_2_O, the appearance of two distinct peaks for each dye indicates the coexistence of monomers and dimers. It should be noted that the transition from mostly monomers to dimers is generally observed at concentrations lower than 0.05 mM.^9,50^

**Figure 7:**
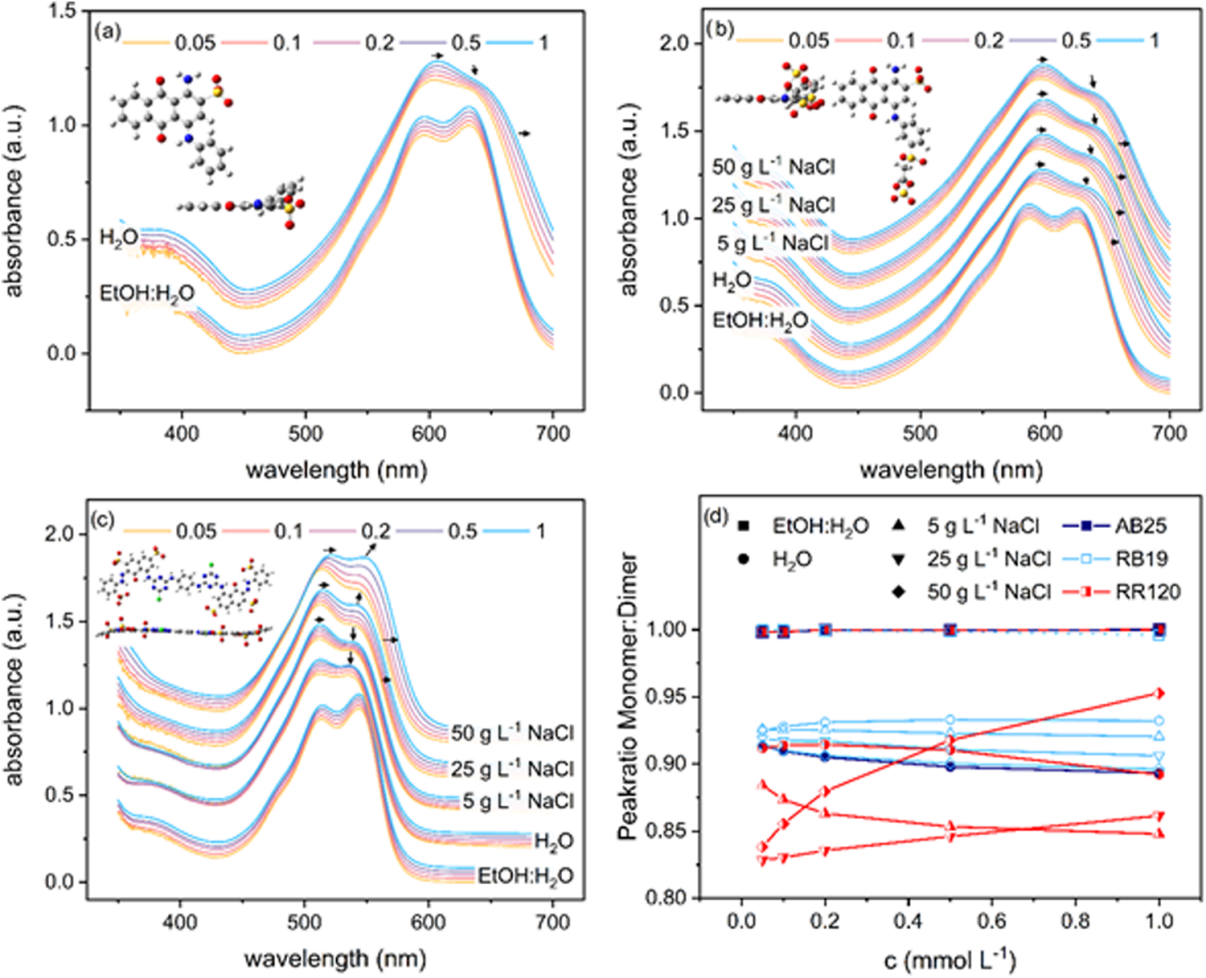
Absorbance curves of AB25 (a), RB19 (b), and RR120 (c) recorded at different dye concentrations (0.05, 0.1, 0.2 0.5, and 1 mmol L^–1^) in a 70 vol % EtOH mixture, in deionized H_2_O, and in NaCl solution at 5, 25, and 50 g L^–1^ in water. The arrows indicate the direction and extent to which the peak changes, with respect to the lowest dye concentration examined for each solvent composition. The molecular structure of the dyes was obtained by DFT calculation from a previous work.^41^ (d) Shows the peak height ratio of monomer to dimer calculated from the absorbance at fixed wavelengths for each dye as a function of concentration.

The peak at the higher wavelength is assigned to the monomer species and the peak at the lower wavelength to the formation of dimers, the so-called H-dimers.^51^ There are no significant differences in the proportion of monomer to dimer peaks, and no broadening is observed with increasing concentration in EtOH/H_2_O. This is further illustrated in Figure 7d, where the normalized ratio of monomer to dimer is plotted against dye concentration. A change in the absorbance spectra is observed when the dyes are dissolved in deionized water compared to EtOH/H_2_O. This is due to the different polarization effect in water and ethanol–water mixtures as the dielectric constant changes.^24,52^ In the case of AB25 (Figure 7a), the dimer peak becomes more prominent at higher dye concentrations, indicating an increase in the number of dimer species relative to the monomers. A slight red shift is explained by the formation of higher-order aggregates and is observed for other dyes.^14^ The RB19 (Figure 7b) absorbance spectra follow the same trend, but the changes are less significant. This may be a consequence of the anchor group, which carries an additional anionic center and makes the molecule less planar, hindering self-association sterically and chargewise. For RR120 (Figure 7c), no peak broadening is observed, but only an increase in the dimer peak, indicating the formation of dimers but not higher-order aggregates.

Self-association is promoted by the addition of NaCl to the dye solutions. In the case of AB25, the addition of 5 g L^–1^ NaCl causes flocculation, indicating a strong tendency toward aggregation. The addition of NaCl to RB19 solutions results in an increase in the dimer peak and a slight shift of the spectra toward higher wavelengths. For RR120, the addition of 5 g L^–1^ NaCl strongly favors the formation of dimers, which becomes more pronounced at higher dye concentrations. At NaCl concentrations of 25 and 50 g L^–1^, a simultaneous increase in the monomer peak position and a shift and broadening of the peak to higher wavelengths are observed. Similar changes in the absorbance spectra of RR120 were reported earlier and attributed to the formation of higher-order aggregates in addition to the formation of dimers,^53^ resulting in an increase of the formal monomer–dimer ratio with increasing dye concentration, as shown in Figure 7d. This study indicates that the aggregation tendency of dyes in water is higher for AB25 and RR120 compared to that of RB19. With the addition of NaCl, AB25 starts to flocculate, and RR120 forms dimers and higher-order aggregates, showing high sensitivity to the addition of NaCl. In contrast, RB19 appears to be less sensitive to NaCl as only small changes in the peak ratio are observed (Figure 7d).

### Influence of the Molecular Structure on Dye Aggregation and Sorption Behavior

Differences in aggregation behavior are closely related to the molecular structure of the dyes. As is well documented, the number and size of anionic groups have been shown to influence dye self-association.^54^ For instance, AB25 is a small anthraquinone dye that bears a single anionic group. Its planar structure promotes π–π interactions and stacking, and the electrostatic repulsion from the single anionic group can be effectively screened even at low NaCl concentrations, leading to flocculation with only 5 g L^–1^ NaCl.

In contrast, the anthraquinone dye RB19 exhibits a reduced propensity for aggregation. This is attributed to the presence of reactive anchor groups, which introduce steric hindrance and thereby inhibit aggregation. The second anionic group in RB19 appears to have limited impact, as evidenced by RR120, which possesses six anionic centers but still begins to aggregate at low NaCl concentrations. This behavior is attributed to its large, planar molecular structure, which strongly favors stacking interactions.

The molecular structure of the dye also plays a significant role in its sorption behavior. In the Langmuir region of the sorption isotherm, dye uptake is primarily limited by the accessibility of dye molecules to cationic groups located within the slit pores of cellulose fibers, as discussed in a previous study.^41^ However, the study did not conclusively determine how the dye’s charge or charge distribution influences the sorption process.

At higher dye concentrations, where aggregation of the dye molecules becomes evident, sorption is no longer confined to the cationic sites and also occurs on noncationized regions of the fiber. It can be concluded that the propensity of dye molecules to aggregate becomes the predominant factor for dye uptake on the fiber.

### Transition of the Sorption Stage to the Fixation Stage

In Figure 8, the dye exhaustion curves of the reactive dyeing experiment on cationized cellulose (salt free) and on noncationized cellulose (+50 g L^–1^ NaCl) are shown. The salt-free process (Figure 8a) is characterized by fast sorption rates and high neutral exhaustion (i.e., prior to addition of alkali) for all dyes due to electrostatic attraction,^33^ while in a conventional process (Figure 8b) only dyes with high affinity (i.e., RR120) show substantial dye uptake at the end of the sorption stage.^55^

**Figure 8:**
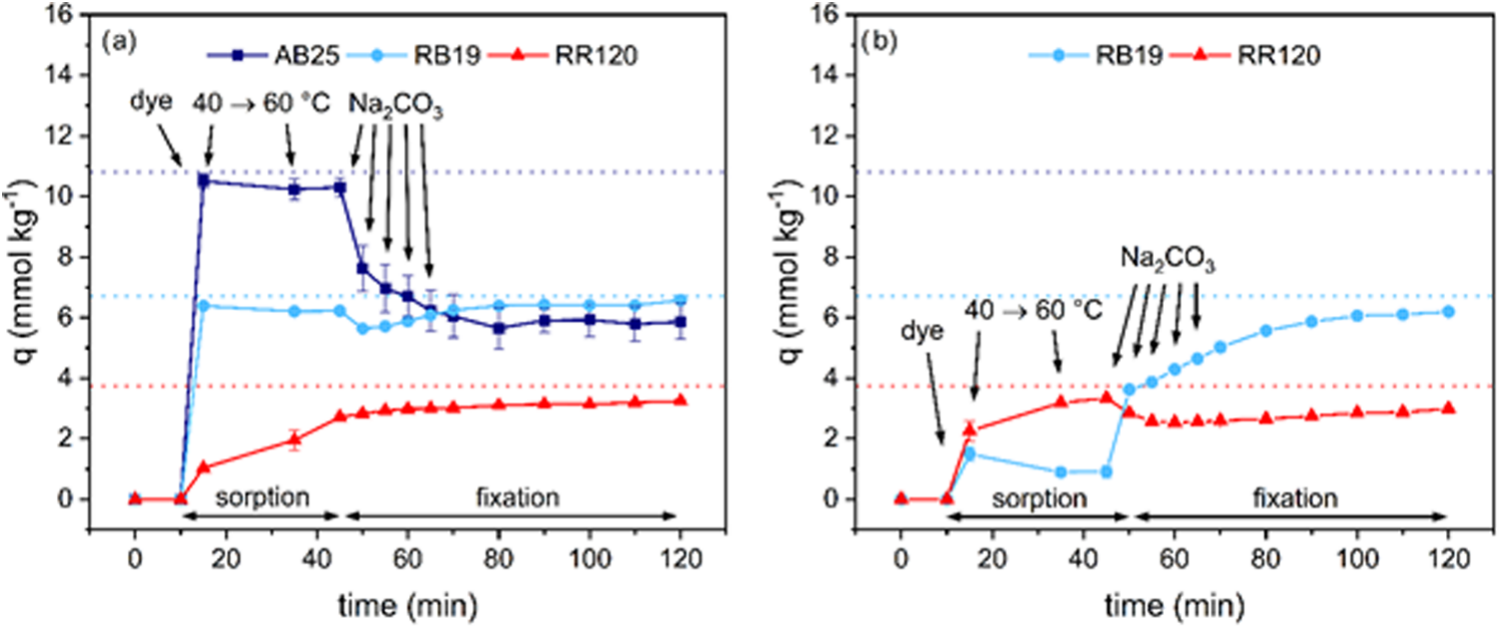
Dye uptake vs time profile for the salt-free reactive dyeing experiments on the cationized jersey and the conventional reactive dyeing experiments with 50 g L^–1^ NaCl on the untreated jersey. The dashed line indicates complete dye exhaustion and the appearance of a colorless dyebath.

The fixation stage is initialized by addition of sodium carbonate, which results in deprotonation of cellulose’s hydroxy groups and the formation of nucleophilic cell–O^–^ centers. This results in two opposing effects on dye exhaustion. First, the fiber becomes more anionic and electrostatic repulsion of anionic molecules is increased, resulting in desorption of dye molecules^56^ and the approach of a new equilibrium at lower sorption levels, which is seen best for AB25 (note that there is no covalent fixation of AB25). Conversely, covalent bonding of reactive dyes to nucleophilic centers continuously disrupts the equilibrium and dyes from the solution adsorb onto the fiber to maintain it, thereby increasing dye exhaustion.^57^ Consequently, both effects perturb the sorption equilibrium, but the contribution of each is different for the specific dye and for cationized and noncationized cellulose. While RB19 shows desorption after addition of alkali in the salt-free dyeing process (Figure 8a), substantial dye exhaustion in the conventional process begins only when alkali is added (Figure 8b). For RR120, minor desorption is observed in the conventional process on addition of alkali (Figure 8b) but is not observed for the salt-free dyeing (Figure 8a).

In Table 2, selected parameters of the dyeing experiments are reported. While the final exhaustion for all reactive dyes is above 80%, major differences are observed in fiber rinsing with water and in extraction of unfixed dye with DMF/H_2_O containing 50 g L^–1^ NaCl. The final rinsing steps with water after the dyeing process resulted in dye wash off from the conventionally dyed samples, and there was also some leaching of dye in the extraction step. In contrast, there was little wash off of dye with water from the cationized fibers, but there was dye leaching during the extraction step. The lack of dye removal only with water from the cationized samples is attributed to the ionic interactions between the dye and fiber. The proportion of covalently bound dye is higher for RB19 in the salt-free dyeing process compared to RR120, which exhibited higher fixation in the conventional dyeing. The levelness (uniformity) of both salt-free and conventional reactive dyeing is comparable, and only minor color differences (Δ*E*) are observed with a Δ*E* around 1.

**Table 2:** Parameters of the Reactive Dyeing Experiment for the Cationized (-cat) and the Conventional (-conv) Dyed Jerseys^a^

sample	neutral exhaustion	final exhaustion	dye washed out	dye extracted	dye covalent bond	*K*/*S*	Δ*E*
AB25-cat	95 ± 3	54 ± 5	5 ± 2	53 ± 1		6.7 ± 0.3	<0.8
RB19-cat	93 ± 1	98 ± 1	0.5 ± 0.1	11.5 ± 0.1	86 ± 1	3.4 ± 0.12	<0.7
RR120-cat	73 ± 3	87 ± 2	0.8 ± 0.2	41 ± 1	45 ± 2	8.7 ± 0.5	<1
RB19-conv	14 ± 3	92 ± 1	22 ± 1	0.3 ± 0.4	71 ± 1	2.7 ± 0.2	<1.1
RR120-conv	89 ± 2	80 ± 1	28 ± 1	1.3 ± 1.3	50 ± 2	2.6 ± 0.2	<1.2

a The values for exhaustion, washout, and extraction are in percent and refer to the initial amount of dye, and *K*/*S* and Δ*E* are dimensionless (further details on the calculation can be found in the Supporting Information).

It should be noted that the dyeing conditions employed in this work do not match the supplier-recommended temperature and alkali levels for the individual dyes. However, the objective was to highlight the difference in dye sorption/fixation profiles between cationized and noncationized fibers for different dye types, and thus the same set of conditions were employed in all experiments.

## Conclusions

The sorption of anionic dye molecules on cationized cellulose fibers is currently understood to follow a Langmuir isotherm, with dye uptake reaching a plateau once all sorption sites are saturated.^16,39^ While this holds true at high levels of dye exhaustion or in diluted dye solutions, the sorption isotherm changes when examined at higher residual dye concentrations. In this work, the sorption mechanism of anionic dye molecules on cationized viscose fibers was investigated as a function of dye and NaCl concentrations.

At low dye concentrations, dye sorption occurs due to strong ionic interaction between anionic dye molecules and cationic centers on the fiber, and a Langmuir plateau is observed. This plateau is maintained in the sorption isotherms until the dye concentration is high enough to induce successive self-association of dye molecules and the formation of aggregates, resulting in an S-shaped rise in dye uptake as a consequence of the lower solubility of aggregates. This secondary dye uptake is attributed to nonionic (and noncovalent) interactions between the dye and fiber. The discussed sorption mechanism is highly dependent on the tendency of dye molecules to aggregate. For planar dye molecules such as AB25 and RR120, a higher tendency for dye aggregation was observed, which significantly increased the dye uptake. However, for RB19, which has the same anthraquinone chromophore as AB25, dye aggregation is sterically hindered due to the reactive anchor group. This is further supported by a decrease in RB19 sorption levels once NaCl is present in the dye solution due to competitive ion exchange.

In order to provide a mathematical description of the sorption data, an adapted Langmuir–Sips isotherm is proposed. This isotherm merges into a Freundlich isotherm at higher concentrations and shows an excellent correlation. The fundamental understanding of the existence of the nonionic sorption mechanism on cationized cellulose is of technical importance, especially in the context of industrial salt-free reactive dyeing. The dye concentrations under investigation are at the lower end of those typically employed in industrial processes. Therefore, the transition from the sorption phase to the fixation phase was studied on a cationized cotton jersey. It was observed that the addition of alkali, in this case Na_2_CO_3_, disrupts the sorption equilibria due to ionization of hydroxy groups and fixation of dye molecules. The former results in desorption of dye molecules with low affinity, and the latter increases dye uptake due to formation of covalent bonds between the dye and fiber. The effect of both is different for the dyes investigated and the cationized and noncationized jerseys, highlighting the importance of readapting dyeing recipes for cationized cellulose.

Future research should investigate the influence of dye molecular structure on sorption-related processes, such as intraparticle diffusion, to enhance the understanding of dye–fiber interactions and the underlying causes of uneven dyeing. Furthermore, a more comprehensive understanding of the impact of dye structure and concentration^58^ on salt-free reactive dyeing is required in order to optimize this process and to develop salt-free reactive dyeing recipes.

## Supplementary Material



## Notes

Details on the calibration of the nitrogen analyzer and the calculation of nitrogen content from the calibration; sample preparation for nitrogen analysis using the 3D-printed punch tool; cationization reaction; and equations and calculations for neutral exhaustion, final exhaustion, dye washed out, dye extracted, dye covalent bond, and Δ*E* (PDF)

## Abbreviations

CHPTAC: 3-chloro-2-hydroxypropyl*-N*,*N*,*N*-trimethylammonium chloride
C.I.: color index
AB25: acid Blue 25
RB19: reactive Blue 19
RR120: reactive Red 120
IEX: ion exchange
