# Supplementary material for: Effect of Dye Aggregation on the Sorption Behavior of Anionic Dyes onto Cationized Cellulose Fibers
Source: Langmuir. 2025 Jul 8;41(28):18766–78. doi: 10.1021/acs.langmuir.5c02070 (PMC12288074; doi:10.1021/acs.langmuir.5c02070)
Supplement: Supplementary file 1 [file la5c02070_si_001.pdf]

Supporting Information

Effect of dye aggregation on the sorption behaviour of anionic dyes onto cationised cellulose fibres

Felix Netzer, Amalid Mahmud-Ali, Avinash P. Manian, Thomas Bechtold, Tung Pham*

Research Institute of Textile Chemistry and Textile Physics, University of Innsbruck, Hoechsterstrasse 73, 6850 Dornbirn, Austria

*Corresponding author:

Tung Pham, email: Tung.Pham@uibk.ac.at

Number of pages: 5

Number of figures: 3

Number of schemes: 0

Number of tables: 0

Table of Contents

Calibration of the Nitrogen Analyser………………………………………………S-2

Sample Preparation for Nitrogen Analysis……………………………………….S-3

Cationisation of Cellulose………………………………………………………….S-3

Transition of the Sorption Phase to the Fixation Phase………………………….S-4

References……………………………………………………………………….S-5

Calibration of the Nitrogen Analyser

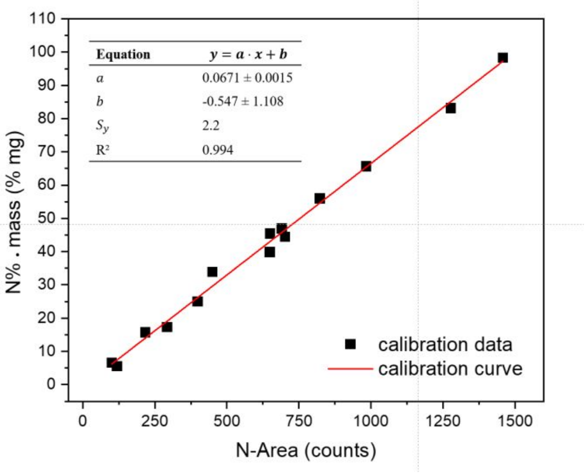

Figure S1. Calibration curve for Nitrogen Analysis.

The instrument was calibrated using carefully measured amounts of aspartic acid ranging from 0.49 to 9.35 mg. To construct the linear regression curve, the peak area of the signal was plotted against the product of nitrogen content (nitrogen content aspartic acid =10.52 wt%) and sample mass. The nitrogen content of the fibre was then calculated using equation S1.

$$N\% = \frac{(N_{area}^{sample} - N_{area}^{untreated}) \cdot a + b}{m_{sample}} \qquad \text{(S1)}$$

$N\%$	Nitrogen content of the sample (%)
N_{area}^{sample}	Nitrogen area from the sample (counts)
$N_{area}^{untreated}$	Nitrogen area from the untreated reference (counts)
a	slope of regression curve (% mg)
b	intersection of regression curve (% mg)
m_{sample}	sample mass (mg)

Sample Preparation for Nitrogen Analysis

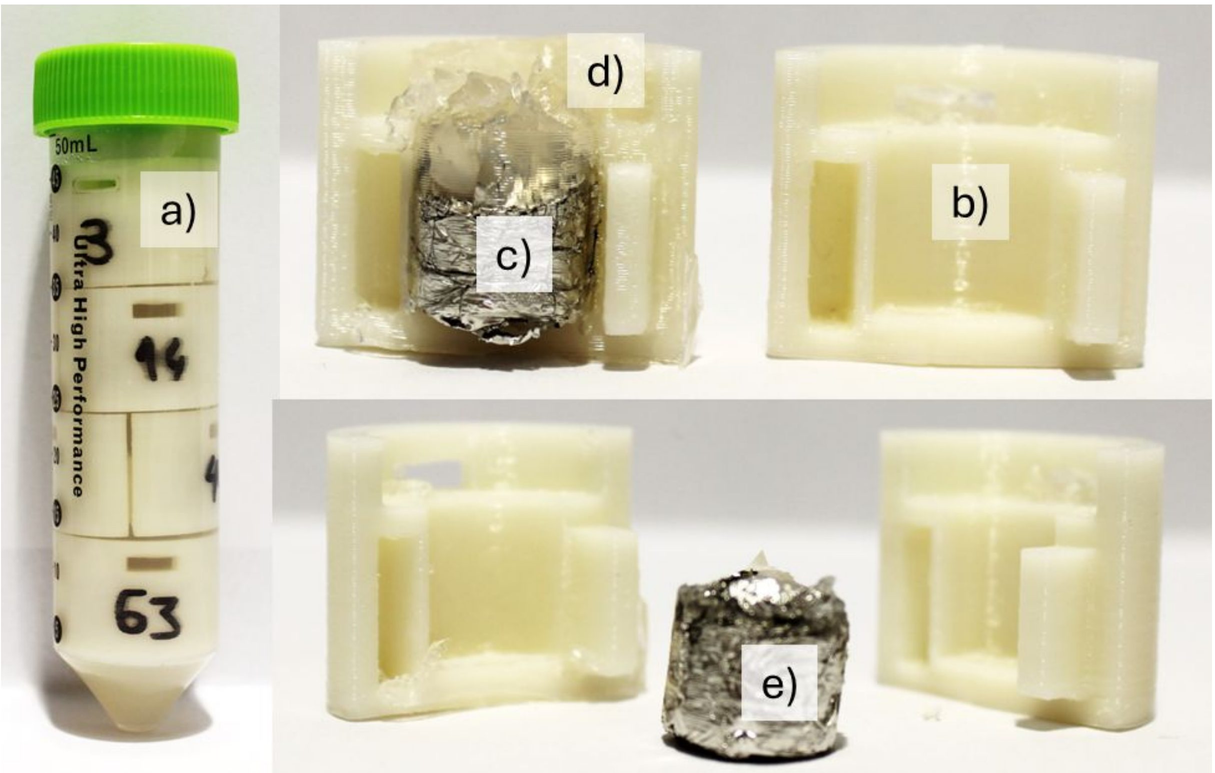

Figure S2. Four punch tools are stacked in the centrifuge tube (a). The punch tool consisted of two halves (b) which were separated during sample recovery (c). Excess agar gel (d) was removed and the recovered sample tablet is shown in e).

The fibres (≈ 350mg) were wrapped in tinfoil and pressed into tablets using a 3D printed punch tool (Figure S2). A needle was used to make holes in the tablet to allow air to escape and agar gel to fill the voids. The 3D printed punch tool with the tablet inside was then placed in a centrifuge tube and covered with 95°C hot 2.5% agar gel. A glass rod was used to compress the tablet and remove most of the air. A total of four tools could be stacked in the centrifuge tube. Holes in the bottom of the tools allowed the exchange of gas and gel. The tube containing the hot gel was then placed in a centrifuge and centrifuged at 4000 G for 10 min. The samples were allowed to cool before the tablets were recovered.

Cationisation of Cellulose

The reaction of CHPTAC with Cellulose under alkaline condition is well documented in literature (i. e. Hashem et al.[1]). The reaction is shown in Figure S3, whereby CHPTAC reacts with Cellulose via the formation of an epoxide as intermediate. The yield of the reactions depends on the amount of alkali, the liquor ratio and the procedure. For exhaust procedure with

liquor ratio 1:20 the expected yield is around 10 % and for cold pad batch procedure the yield is expected to be around 30 %.

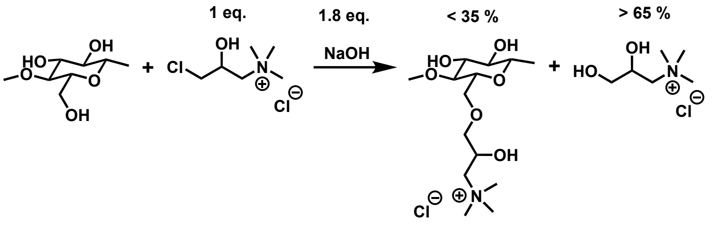

Figure S3. The cationisation reaction of cellulose with CHPTAC under alkaline conditions.

Transition of the Sorption Phase to the Fixation Phase

Table 2 of this work gives relative values for neutral exhaustion, final exhaustion, washed out dye, extracted dye and covalent bond dye. These values are calculated in relation to the initial amount of dye present in the dye bath.

The neutral exhaustion is calculated from the dye uptake after 45 minutes before the addition of alkali according to equation S2. The final exhaustion is calculated from the dye uptake at the end of the dyeing experiment after 120 min according to equation S3. The amount of dye washed out is calculated from the total amount of dye removed during the five washing steps (equation S4). The amount of dye extracted is calculated from the amount of dye removed during the dye extraction experiments (equation S5). The percentage of fixation is calculated from the amount of dye on the fibre after washing and dye extraction (equation S6).

$$E_{neutral}(\%) = \frac{q_{45\,min}}{q_{100\%}} \cdot 100\% \qquad \text{(S2)}$$

$$E_{fixation}(\%) = \frac{q_{120\,min}}{q_{100\%}} \cdot 100\% \qquad \text{(S3)}$$

$$Wash\ (\%) = \frac{q_{washed\ out}}{q_{100\%}} \cdot 100\% \qquad \text{(S4)}$$

$$Ext\ (\%) = \frac{q_{extracted}}{q_{100\%}} \cdot 100\% \qquad \text{(S5)}$$

$$F(\%) = \frac{q_{120\,min} - q_{washed\ out} - q_{extracted}}{q_{100\%}} \cdot 100\% \qquad \text{(S6)}$$

$E(\%)$ Dye Exhaustion (%)

$Wash$	Dye washed out (%)
$Ext(\%)$	Dye extracted (%)
$F(\%)$	Dye Fixation (%)
$q_{45,120}$	Dye uptake after 45, 120 min (mmol kg^{-1})
$q_{washed\ out}$	Dye removed from the fabric during washing (mmol kg^{-1})
$q_{extracted}$	Dye removed from the fabric during dye extraction (mmol kg^{-1})
$q_{100\%}$	Theoretical dye uptake of 100 % from the dye bath (mmol kg^{-1})

The colour difference ΔE is calculated from the average L*, a* and b* values from the colour photometric measurement of the six positions on the fabric according to equation S7.

$$\Delta E = \sqrt{\left(L_i^* - L^*\right)^2 + \left(a_i^* - a^*\right)^2 + \left(b_i^* - b^*\right)^2} \qquad \text{(S7)}$$

ΔE	Colour Difference
L_i^*	Lightness value at position i ($i = 1,2,3,4,5,6$)
a_i^*	value of red-green axis at position i ($i = 1,2,3,4,5,6$)
b_i^*	value of yellow-blue axis at position i ($i = 1,2,3,4,5,6$)
L^*	average Lightness value of six positions
a^*	average value of red-green axis of six positions
b^*	average value of yellow-blue axis of six positions

References

(1) Hashem, M.; Hauser, P.; Smith, B. Reaction Efficiency for Cellulose Cationization Using 3-Chloro-2- Hydroxypropyl Trimethyl Ammonium Chloride. *Textile Res J* **2003**, *73* (11), 1017–1023. https://doi.org/10.1177/004051750307301113.
